# Effects of Two Months of Very Low Carbohydrate Ketogenic Diet on Body Composition, Muscle Strength, Muscle Area, and Blood Parameters in Competitive Natural Body Builders

**DOI:** 10.3390/nu13020374

**Published:** 2021-01-26

**Authors:** Antonio Paoli, Lorenzo Cenci, PierLuigi Pompei, Nese Sahin, Antonino Bianco, Marco Neri, Massimiliano Caprio, Tatiana Moro

**Affiliations:** 1 Department of Biomedical Sciences, University of Padua, 35131 Padua, Italy; antonio.paoli@unipd.it; 2 Research Center for High Performance Sport, UCAM, Catholic University of Murcia, 30107 Murcia, Spain; 3 Brain, Mind and Computer Science Doctoral Program, University of Padua, 35131 Padua, Italy; lorenzo.cenci@studenti.unipd.it; 4 Unit of Pharmacology, School of Pharmacy, University of Camerino, 62032 Camerino, Italy; pete.pompei@unicam.it; 5 Faculty of Sport Science, Ankara University, 06830 Ankara, Turkey; nesesahin@ankara.edu.tr; 6 Department of Psychology, Educational Science and Human Movement, Sport and Exercise Sciences Research Unit, University of Palermo, 90128 Palermo, Italy; antonino.bianco@unipa.it; 7 Department of Human Sciences and Promotion of the Quality of Life, San Raffaele Roma Open University, 00166 Rome, Italy; neri@cervia.com (M.N.); massimiliano.caprio@uniroma5.it (M.C.); 8 Laboratory of Cardiovascular Endocrinology, IRCCS San Raffaele Pisana, 00163 Rome, Italy

**Keywords:** ketogenic diet, strength, resistance training, health

## Abstract

**Background**: Ketogenic diet (KD) is a nutritional approach that restricts daily carbohydrates, replacing most of the reduced energy with fat, while maintaining an adequate quantity of protein. Despite the widespread use of KD in weight loss in athletes, there are still many concerns about its use in sports requiring muscle mass accrual. Thus, the present study sought to investigate the influence of a KD in competitive natural body builders. **Methods**: Nineteen volunteers (27.4 ± 10.5 years) were randomly assigned to ketogenic diet (KD) or to a western diet (WD). Body composition, muscle strength and basal metabolic rate were measured before and after two months of intervention. Standard blood biochemistry, testosterone, IGF-1, brain-derived neurotrophic factor (BDNF) and inflammatory cytokines (IL6, IL1β, TNFα) were also measured. **Results**: Body fat significantly decreased in KD (*p* = 0.030); whilst lean mass increased significantly only in WD (*p* < 0.001). Maximal strength increased similarly in both groups. KD showed a significant decrease of blood triglycerides (*p* < 0.001), glucose (*p* = 0.001), insulin (*p* < 0.001) and inflammatory cytokines compared to WD whilst BDNF increased in both groups with significant greater changes in KD (*p* < 0.001). **Conclusions**: KD may be used during body building preparation for health and leaning purposes but with the caution that hypertrophic muscle response could be blunted.

## 1. Introduction

Ketogenic diet (KD) is a nutritional approach based on a reduced intake of carbohydrates (less than 20/30 g per day or 5% of total energy) [1,2,3], a high fat content and an adequate level of proteins, the latter generally close to or slightly higher than the Italian recommended daily intake (LARNs recommendation) of 0.9 g per kg of body weight [4]. The metabolic advantage induced by KD is represented by the drastic reduction in carbohydrates, which force the body to primarily use fat as a fuel source as demonstrated by the decrease of the respiratory exchange ratio (RER) [5,6].

Glucose is a fundamental energy source for the central nervous system, which is protected by the blood-brain barrier not allowing the passage of free fatty acids. After a few days of KD, there is a drop in glucose availability due to reduced glycogen stores and thus an alternative energy source is required [7,8]. This energy is supplied by ketone bodies (KBs): acetoacetate (AcAc), 3-hydroxybutyrate (BHB) and acetone. KBs are generated through a process called ketogenesis from acetyl-CoA that occurs mainly in the mitochondrial matrix of the liver. Although created in the liver, this organ is unable to utilize KB due to a lack of the succinyl-CoA: 3-CoA transferase enzyme, which is required to convert acetoacetate to aceto-acetyl-CoA. KBs are thus released into the extrahepatic bloodstream and can be used by other tissues as energy source, by converting BHB into acetoacetate and then into aceto-acetyl-CoA, which can be further transformed into two acetyl-CoA molecules and enter the Krebs cycle [9]. At the end of this process, the energy produced from KBs is greater compared with glucose [2,10]. Initially, the necessary amount of acetyl-CoA is supplied by hepatic glycogenolysis and gluconeogenesis, but after four–seven days of KD, lipolysis of adipose tissue becomes the main source. In the long term, this adaptation translates into a reduction in the respiratory ratio which represents the metabolic switch towards a greater reliance on fatty acids [6,11].

KD is a well-proven approach to promote weight loss [2], and it has recently been used as a valuable therapeutic option to treat metabolic disorders, cardiovascular disease and type 2 diabetes [12]. Its role in sport performance is still controversial, with some authors experiencing favorable effects [3,13] whilst other discourage the use of such dietetic approach, at least in endurance sports [14,15,16]. For athletes who compete in weight-category sports, KD could be a safe weight loss method, not compromising performance, therefore it may represent a legitimate and important tool for athletes. Paoli et al., for instance, demonstrated that 30 days of KD decreased body weight and body fat without negative effects on strength in high level gymnasts [13]. However, it might seem counterproductive for those athletes who seek maximum muscle hypertrophy [17], as several studies have shown no accretion of muscle mass during the ketosis phase [13,18,19,20]. Despite this, numerous bodybuilders use KD without a justified reason; however, no studies have yet explored its role in this particular sport category [21,22,23]. Professional bodybuilders’ goals are to keep a perfect balance between muscle size and body fat, in order to obtain the most accurate symmetry and muscular proportion. To achieve their objectives, bodybuilders undergo a cycle of different training intensities in combination with various dietetic regimen in order to increase muscle mass during the “off-season” and reduce fat mass during competition preparation [21]. In this context, KD seems to be a useful diet to reduce fat mass, but its role in preserving athletic performance and muscle hypertrophy in physique athletes is still poorly investigated.

The high intensity and frequency of training employed by bodybuilders during their contest preparation may also induce muscle damage [24,25,26], which could ultimately result in a chronic inflammatory status, with increased level of IL-6, IL-1 and TNF-α [27]. On the other hand, a weight loss program can increase stress, anxiety and negatively affect athlete’s mood [28]. The brain-derived neurotrophic factor (BDNF) is a protein associated with major depressive disorders and stress situations [29,30]. It has been shown that KD may have positive effects on inflammation [31,32,33] and can reduce depression-like behaviors [34] in both animals and human models, but no data are available about its effect on athletes such as bodybuilders.

The aim of the present study is to evaluate the effects of the ketogenic diet on body composition, muscle mass, strength and some blood parameters mainly related to lipid profile, hormonal (i.e., IGF-1) and inflammatory status (i.e., IL-6, TNFα and IL-1β) in competitive body building athletes. Basal metabolism and respiratory quotient were also assessed in order to monitor the metabolic adaptation to KD.

## 2. Materials and Methods

**Figure 1:**
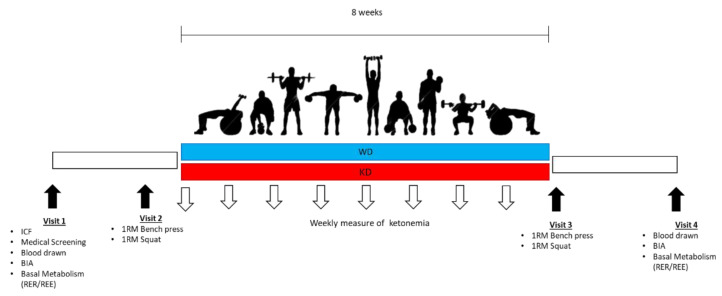
Study design. ICF, Informed consent form signed; BIA, Bioelectrical Impendence Analysis; REE, Basal metabolism; RER, respiratory quotient; 1 RM, 1 repetition-maximum test; WD, Western diet, KD, Ketogenic Diet.

This study is a randomized controlled parallel study (Figure 1). After signing the informed consent, the participants were invited to refer to the Exercise Nutrition and Physiology laboratory of the Department of Biomedical Sciences of the University of Padova. During the first visit, the participants underwent a medical screening to ensure eligibility for the study and a food interview to gather information on the participants’ dietary habits. Subjects were examined after overnight fasting for blood sample collection, followed by body composition measurements via bioimpedance analysis (BIA) and basal metabolic rate assessment via indirect calorimetry. In a second visit, participants underwent maximal strength tests (1-RM) for bench press and squat exercises. During the second visit, all subjects received a personalized diet protocol, to be followed for eight weeks; adherence to the dietary regimen and ketonemia were monitored weekly through a portable device. Weekly results of blood BHB were sent to the research team: a value under 0.5 mmol/L was selected as non-adherence index and used to exclude subjects from the analysis. All participants maintained BHB levels over the defined limit and were included in the final analysis. Participants kept their own training routine, which was personalized and different for all participants. Athletes took part in the study in a period away from the competition phase: workouts had a daily schedule and were divided by muscle groups, and training sessions included exercises mainly aimed at increasing strength and muscle mass. After eight weeks subjects repeated the strength tests and body composition analysis and basal metabolism were reassessed 72 h after the last workout blood sampling

### 2.1. Subjects 

Nineteen male athletes (age 27.42 ± 10.54 years; BMI 26.80 ± 1.91 kg/m^2^; lean mass 88.62 ± 2.81%) were recruited from the sports centers of Emilia-Romagna and Veneto through advertising direct to coaches. Subjects aged between 20 and 40 years old were included in the study if they had at least five years of training experience and were competing in a recognized body building category. Exclusion criteria were use of steroids, chronic use of any medication, metabolic disorders or any other clinical problems that could be aggravated by the study procedures. Use of steroids was excluded by coaches’ interview, and as athletes had officially enrolled in natural body building categories. Table 1 shows the anthropometric characteristics at baseline. Before any procedures, all participants signed the informed consent for data collection approved by the ethical committee of the Department of Biomedical Sciences, University of Padova (HEC-DSB 11/19), according to the current Declaration of Helsinki. At the end of the first screening, subjects were randomly assigned to these experimental groups: ketogenic diet (KD; *n* = 9) or control western diet (WD; *n* = 10). The study was retrospectively registered at clinical trials.gov as NCT04629365.

### 2.2. Measurements

Subjects underwent blood sampling after an overnight fast for blood glucose, insulin, total cholesterol, HDL cholesterol, LDL cholesterol, triglycerides, aspartate transaminase (AST), alanine amino transferase (ALT), total testosterone, insulin growth factor 1 (IGF-1), brain-derived neurotrophic factor (BDNF), interleukin-1 (IL-1); interleukin-6 (IL-6) and tumor growth factor (TNF-α). All the analysis was performed by an accredited and certified laboratory.

Body composition assessment was performed by Bioelectrical Impendence Analysis (Akern mod. STA/BIA 101/S, Pontassieve, FI, Italy). Subjects were asked to lie down and rest for about three–five min in order to allow a balanced redistribution of body fluids. Four skin electrodes were then applied, one on the back of the hand, one on the metacarpal-phalangeal joint of the third finger, one on the dorsum of the ipsilateral foot at the metatarsal-phalangeal joint of the third finger and one on the ankle joint. Using dedicated analysis software (Akern, Body Pro, Pontassieve, Italy) we obtained the values of lean mass (FFM) and fat mass (FM).

Basal metabolism (REE) and respiratory quotient (RER) were measured via indirect calorimetry. Before each procedure, the respiratory gas analyzer (Max Encore 29 System, Vmax, Viasys Healthcare, Inc., Yorba Linda, CA, USA) was calibrated using a special syringe. During the test, participants lay on a cot in a room that was not too bright, quiet and with a temperature of about 24 °C. During the test, participants were asked to breathe regularly in a silicone mask that allowed the analysis of respiratory gases. Data was collected for 30 min, however, only the last 20 min were taken into account for data analysis. REE was calculated starting from oxygen consumption (VO_2_), based on the Weir equation, while RER was derived from the ratio between the production of VO_2_ and carbon dioxide (VCO_2_).

Muscle strength was assessed during the second visit. 1-RM test was performed during the squat exercise for the lower limbs’ strength, and during the bench press exercise, for the pectorals as previously described [35].

Subjects were requested to refrain from any physical exercise other than normal activities of daily living for at least 48 h prior to testing. Repetition maximum testing was performed according to the guidelines of the National Strength and Conditioning Association [36]. Briefly, subjects performed a general warm-up prior to testing, followed by a specific warm-up set for each exercise consisting of 5 repetitions at 50% of an estimated 1-RM followed by one to two sets of 2–3 repetitions at a load corresponding to 60–80% of 1-RM. Subjects were then asked to gradually reach the load with which they could perform a maximum of one repetition while maintaining the correct realization of the movement: for the bench press the barbell had to touch the chest and return to full arm extension at each repetition without bouncing, and during the squat each movement had to be completed with thighs parallel to the floor

### 2.3. Diet Protocols

The diet was formulated by a dietary team on the basis of the food interview that took place during the first screening visit and in consideration of the specific caloric energy and macronutrient needs for body-building athletes [37] using software for the elaboration of diets (Dieta Ragionata 7.0).

The two diet regimens were isocaloric and included the same amount of protein per kg of body weight. The caloric intake of the dietary patterns provided was calculated by assigning an energy expenditure of 45 kcal/kg of muscle mass, while the protein intake was maintained at 2.5 g/kg/body weight as suggested by Apong in 2019 [37]. The two protocols differed in the distribution of fats and carbohydrates; the latter were kept below 5% daily (less than 50 g/day) in the KD group while they represented 55% of the caloric intake in the WD group. The caloric and macronutrient distribution are shown in Table 2. Bodybuilders are known to slavishly follow prescribed diets, normally composed of a restricted and repetitive food regimen [23,38,39]. This condition helped to guarantee the adherence to the prescribed diet in both groups throughout the study. Moreover, blood BHB was regularly checked to avoid exit from ketosis in the KD group.

### 2.4. Statistical Analysis

Data analysis was performed using GraphPad Prism software version 8.4.3 (GraphPad Software, San Diego, CA, USA). Sample size was obtained assuming within subject variability of 30% and a fixed power of 0.8, and an alpha risk of 0.05 for the main variables. The analysis determined that at least eight participants per group were needed to achieve the above parameters. Subjects were randomly allocated in one of the two groups using a using computer generated software.

Results are presented as mean ± SD. After testing for normal distribution with the Shapiro-Wilk W test, a two-way ANOVA for repeated measures was performed to compare the two types of diet through a “time × diet” analysis. Whenever significant differences in values were found, the post-hoc Bonferroni test was used to identify specific intragroup differences. The *p*-value was set at 0.05.

## 3. Results

All the recruited subjects successfully completed the study.

**Figure 2:**
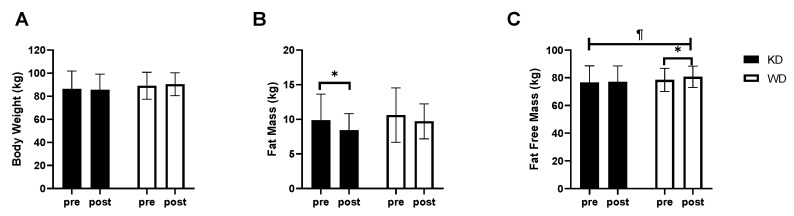
Body composition results after 8 weeks of diet. (**A**) body weight; (**B**) Fat Mass; (**C**) Fat Free Mass. * significantly different from pre-value (*p* < 0.05); ¶ time × diet interaction (*p* < 0.05). KD, ketogenic diet group; WD, western diet group.

Body composition analysis showed that the ketogenic regimen (KD) decreased body weight by approximately 1% (from 86.39 ± 15.42 kg to 85.51 ± 13.62 kg), while the WD determined an increase in weight of about 2% (from 89.04 ± 11.73 kg to 90.37 ± 9.91 kg). Although these differences were not statistically significant (*p* > 0.05), they induced a significant change in body composition (Figure 2). Fat mass significantly decreased only in the KD group (KD: 9.86 ± 3.79 kg to 8.42 ± 2.41 kg, *p* < 0.05 vs. WD: 10.60 ± 3.92 kg to 9.70 ± 2.53 kg) whereas, lean mass significantly increased only in the WD group (KD: from 76.53 ± 12.13 kg to 77.09 ± 11.47 kg vs. WD: from 78.44 ± 8.31 kg to 80.67 ± 7.72 kg, *p* < 0.05) presenting a significant time × diet interaction (*p* = 0.015). In terms of the fat mass and lean mass distribution, it was observed that both groups reduced fat mass (KD: −11.32 ± 7.88% vs. WD: −6.70 ± 10.02%), but the difference was statistically significant only in KD group (*p* < 0.05). The decrease in the percentage of fat mass in the KD group was associated with a significant increase in lean mass (KD: + 1.63 ± 1.49%; *p* = 0.01 vs. WD: +1.20 ± 1.62%; *p* = 0.05).

Strength was increased in both study groups, both in the bench press test and in squat test. There were no significant differences between study groups. As shown in Table 3, the KD group improved their performance in bench press and squats by 4.13% and 3.62%; while the WD group improved by 3.75% and 6.40%, respectively.

Analysis by indirect calorimetry (Table 3) showed a significant increase in REE only in the WD group (WD: + 2.85 ± 1.78%; *p* < 0.05 vs. KD: + 1.99 ± 3.04%). RER revealed a significant time × diet interaction (*p* < 0.0001) with a significant decrease only in the KD group (−3.77 ± 1.86% *p* = 0.02), whilst in the WD group it remained unchanged.

Blood analyses revealed an improvement in the lipid profile only in the KD group (Table 4). Indeed, total cholesterol significantly decreased in the KD group (KD: −3.51 ± 3.72%, *p* < 0.05 vs. WD: −1.59 ± 3.51%) and the HDL component exhibited a significant time × diet interaction (*p* = 0.004), with an increase in the KD group (4.93 ± 3.53%, *p* < 0.05 vs. WD: −1.07 ± 4.46%) and the difference between groups after eight weeks of intervention was statistically different. Triglycerides also presented a significant time × diet interaction (*p* < 0.001), in which lipids significantly decreased in the KD group (−17.44 ± 7.16%; *p* < 0.0001) but not in the WD group (−1.59 ± 5.50%).

**Figure 3:**
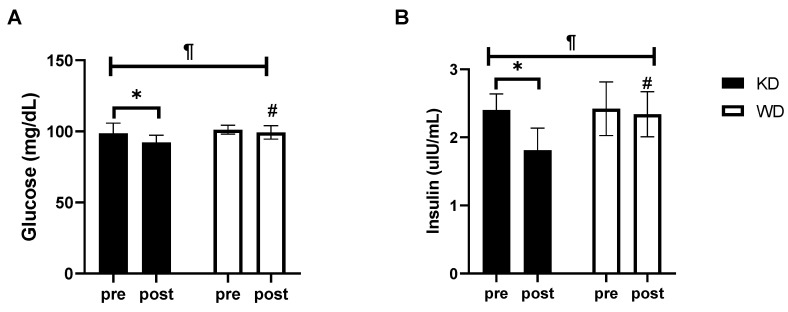
Insulin sensitivity results. (**A**) plasma glucose concentration; (**B**) plasma insulin concentration. * significantly different from pre-value (*p* < 0.05); # significantly different from KD group (*p* < 0.05); ¶ time × diet interaction (*p* < 0.05). KD, ketogenic diet group; WD, western diet group.

Similarly to what was seen for HDL cholesterol, glucose and insulin concentrations also significantly decreased only in the KD group at the end of the eight weeks of treatment (Figure 3). Plasma glucose levels were reduced from 98.67 ± 6.68 mg/dL to 92.22 ± 4.76 mg/dL in the KD group (*p* < 0.001), and from 101.20 ± 3.12 mg/dL at 99.30 ± 4.76 mg/dL in the WD group. Insulinemia decreased from 2.40 ± 1.81 µU/mL to 1.81 ± 0.31 µU/mL in the KD group (*p* < 0.0001) while in the WD group it went from 2.42 ± 0.39 µU/mL to 2.34 ± 0.33 µU/mL.

Transaminase analysis (Table 4) showed no difference for aspartate transaminase (AST), while a significant time × diet interaction (*p* < 0.01) was observed in alanine amino transferase (ALT), whose concentrations decreased significantly only in the KD group (*p* < 0.05) but not in the WD group.

As regards hormone levels, the trend already observed in other studies was confirmed, in that anabolic hormones, such as testosterone and IGF-1 (Table 4), decreased in KD group. A significant time × diet interaction was observed (*p* < 0.05), resulting in a significant decrease in the KD group (total testosterone −10.22 ± 6.95%, *p* < 0.001; IGF-1 −16.43 ± 8.52%, *p* < 0.05) but not in the WD (testosterone total from −2.01 ± 5.45%; IGF-1 −0.18 ± 9.48%).

**Figure 4:**
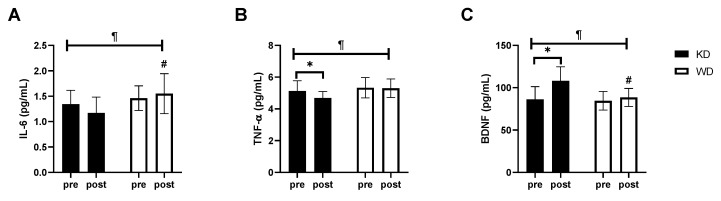
Inflammatory markers and brain-derived neurotrophic factor (BDNF). (**A**) IL-6 concentration; (**B**) TNF-α concentration; (**C**) BDNF concentration. * significantly different from pre-value (*p* < 0.05); # significantly different from KD group (*p* < 0.05); ¶ time × diet interaction (*p* < 0.05). KD, ketogenic diet group; WD, western diet group.

The analysis of inflammatory markers did not reveal any changes in IL-1 (KD: from 0.92 ± 0.14 pg/mL to 0.87 ± 0.10 pg/mL; WD: from 0.93 ± 0.10 pg/mL to 0.96 ± 0.08 pg/mL), while a significant time × diet interaction (*p* < 0.05) emerged for IL-6 and TNF-α (Figure 4). The IL-6 presented an opposite trend in the two groups; in fact it decreased in the KD group by 13.35% and increased in the WD group by 6.52%, resulting in a statistically significant difference between the two groups at the end of the study protocol (KD: 1.17 ± 0.30 pg/mL vs. WD: 1.55 ± 0.39 pg/mL; *p* < 0.05). TNF-α, on the other hand, remained almost unchanged in the WD (from 5.33 ± 0.64 pg/mL to 5.30 ± 0.58 pg/mL), while it significantly decreased in the KD group (from 5.12 ± 0.61 pg/mL to 4.69 ± 0.38 pg/mL; *p* < 0.01).

Finally, BDNF concentrations (Figure 4) significantly increased only in the KD group (+25.84 ± 11.01%; *p* < 0.0001) at the end of the eight weeks of treatment (KD: 108.00 ± 15.68 pg/mL vs. WD: 88.60 ± 10.62 pg/mL; *p* < 0.01).

## 4. Discussion

To our knowledge this is the first study analyzing the effects of a KD on bodybuilders. Our findings confirm several preliminary data on the effects of KD diet on different power sports. KD induced a significant loss of fat mass without affecting muscle performance. Interestingly, fat free mass was maintained throughout the two months of KD despite a significant reduction in blood anabolic hormone concentrations. Moreover, blood lipids, glucose and inflammatory markers were improved in the KD group.

While the effects of KD on fat mass are consistent in all studies evaluating body composition during KD regimens on athletes [13,20,40], the same was not confirmed for lean mass, with some authors showing a catabolic effect of KD on muscle [19,41,42,43], others showing no direct effects [13,44]. Only one study showed a higher increase in lean body mass that was measured after one week of carbohydrate recharge [45]. Muscle mass accrual is obtained under a chronic stimulation of muscle protein synthesis, which is governed by hormonal (IGF-1, testosterone) and transcriptional regulatory pathway (Akt/mTOR). During KD, the reduction in carbohydrates intake leads to a decrease in insulin levels [46]. Insulin is the main regulator of glucose uptake and is regulate by plasma glucose level; as such, lower level of blood glucose caused by the reduction of carbohydrates ingestion, inhibit β-cells insulin secretion [9]. Insulin is also a potent anabolic hormone [47], and a reduction in insulin levels facilitates mobilization from fat stores due to its effects on lipo-synthesis/lipolysis balance; on the other hand, it may inhibit muscle growth pathway. On a transcriptional level, KD seems to be able to increase the phosphorylation of the AMP-activated protein kinase (AMPK) [48], which has a well-known effect on Akt/mTOR pathway’s inhibition [30]. In the present study we were not able to examine the effect of KD on a molecular level, but we observed, as expected, a significant decrease in insulin concentrations, which may play a role in body fat utilization [49]. We also observed an unexpected decrease in testosterone concentration. Most of the studies conducted so far showed a negligible effect of KD on testosterone [50,51], whilst our results showed a drop of ~11%, accompanied by a significant decrease in IGF-1 (~15%). Taken together, the anabolic hormonal response seems to be blunted during the KD regimen, and this may explain the attenuated hypertrophic response that was observed. However, in contrast with the studies that showed a decrease in lean body mass during KD, we observed a preservation of FFM; this is most likely due to the effect of resistance training stimuli and also to the maintenance of an high dietary protein level (2.5 g/BW) [13,14,17,52].

If a low carbohydrate intake has affected muscle gain, it did not alter muscle performance. Strength and power athletes are recommended to maintain a higher carbohydrate intake (3–8 g/kg) [53,54,55] to sustain the intramuscular glycogen stores and engage in greater training volume. It has been observed that during KD regimen muscle glycogen stores can decrease by ~40–50% [56,57]. In a recent study from Chappell et al. [23], the authors observed that bodybuilders with better results during the competition season normally consumed a greater amount of carbohydrates (5.1 g/kg) compared to non-placed athletes. Recently, Vargas-Molina et al., while confirming the maintenance of lean body mass during KD in strength trained women, found a decrease of strength in bench press and squat exercises [20]. On the contrary, despite the above cited guidelines, we [13] and others [58] observed similar improvement in strength expression after KD compared to normal diet groups. Although further studies are required to better explore this aspect, the majority of studies seem to indicate that KD does not impact muscle performance.

Variations in the basal metabolic rate and respiratory quotient parameters confirm the ability of KD to lower the RER, indicating a shift towards lipid metabolism. Lower RER value seems to predict a better ability to handle future body weight [59,60]; this could be an advantage for bodybuilders, who normally undergo to repetitive cycle of bulking and cutting during their carrier. Moreover, REE did not change in the KD group while it was slightly increased in the WD group, suggesting that KD does not negatively affect basal metabolism. The preservation of lean mass observed in the present study certainly contributed to the control of basal metabolism [61]. Additionally, these results suggest that an improvement in resting nutrient oxidation could be one of the main mechanisms by which KD reduces body fat even though the energy intake was similar in the two groups. There is still a debate about the mechanisms underlying the demonstrated greater effects of KD on weight loss in ecological studies. There are some studies pointing out that that the fat loss induced by a ketogenic diet relies only on calorie deficit [62] whilst others suggested that KD induces an increase in REE [52,63]. No significant differences in energy intake between groups were detected in our study. Thus, a possible explanation of the greater fat loss in the KD may rely on increased (not measured in our study) spontaneous physical activity during the daily normal activities as suggested by Hall et al. [64].

Bodybuilders’ training regimen, which unfortunately is sometimes accompanied with the use of self-administrated steroids, may increase LDL concentration, and reduce HDL levels, increasing the risk of cardiovascular diseases [65,66]. In this context, KD may represent a useful tool to control lipid markers. The improvement in lipid profile with KD is well documented in obese and overweight subjects [67,68], whilst the results are still contradictory in athletes, probably due to the difference in the type of training (aerobic or anaerobic activities) adopted in the study design [19,69]. We observed an improvement of TGs, HDL and LDL cholesterol blood concentrations. KD can directly influence endogenous cholesterol synthesis via the reduction of insulin production: insulin is indeed one of the major activators of the HMGCoA reductase, the key enzyme of cholesterol biosynthesis. The concomitant reduction in carbohydrates intake and increase in dietary cholesterol (derived from fat intake) results in a consequent inhibition of endogenous cholesterol production [70]. Moreover, during KD, dietary triglycerides are rapidly metabolized to release glycerol, which can be used in the liver for energy purposes [71].

KD effect on insulin and glycemic control deserves mention. Several researches have demonstrated that KD can reduce glucose and insulin concentration in obese, insulin resistant individuals and diabetics [72,73,74,75]. This effect is not only related to a reduced intake of glucose within the diet, but also to the improvement in insulin sensitivity [12]. In the present study, despite the healthy status of the participants, KD induced a significant drop in blood glucose which remained within normal levels. We also observed a significant reduction in ALT levels. ALT is not only a hepatic enzyme (although non-specific) but it is a critical enzyme for energy homeostasis. During fasting or sustained exercise, glucose can be synthetized from glucogenic amino acids, such as alanine and glutamate. Specifically, ALT can convert alanine into pyruvate to generate glucose via gluconeogenesis [76]. In obese and diabetic patient, elevated ALT activity has been associated with an impaired insulin sensitivity [77,78] suggesting that ALT may play a pivotal role in the pathogenesis of insulin resistance. The reduced ALT levels observed in the KD group may suggest that the use of ketone bodies as an energy source is optimized to reduce the need for amino acids for glucogenic purposes; on the other hand, these results also seem to confirm the improvement in insulin sensitivity, and consequently a reduced risk of developing diabetes or insulin resistance.

Another extremely interesting outcome is the inflammatory response. IL-1β remain unaltered, whilst TNF-α and IL-6 decreased during KD. These cytokines are linked to oxidative stress or inflammation; thus, their reduction could indicate an attenuation of basal inflammatory status. Furthermore, TNF-α and IL-6 concentrations are generally related to insulin levels; in fact, these cytokines play an important role in the pathogenesis of insulin resistance [60,61,62,63]. Intense protocols of resistance training induce muscle damage, which promote an acute inflammatory response and eventually generates oxygen free radicals and lipid peroxidation [64,65]. If the acute rise in IL-6 could be related to training and is indeed a fundamental factor in adapting to exercise stimuli, chronic elevated levels of IL-6 can interact with the STAT3/TLR-4 pathway and decrease insulin sensitivity and display an additional deleterious effect. KD seems to be a useful tool to modulate the inflammatory response, reducing the basal level of proinflammatory cytokines.

Finally, we observed an increase in BDNF blood concentration during KD. BDNF is a molecule of the neurotrophin family involved in trophism and neuronal plasticity, but also in the energy modulation and glucose homeostasis of the central nervous system [79]. A reduction in this protein can be associated with situations of stress, depression, mood disorders, cognitive aspects and other psychological problems [29,30]. An animal model of depression presented reduced level of BDNF [80], and when administrated BDNF seems to display antidepressant effect [81]. Some research has revealed that during weight loss programs subjects may experience depression and negative feelings [28]. However, in humans, KD has been shown to raise BDNF levels, probably via BHB regulation [82], which was also associated with substantial improvement in cognitive function [83]. BHB regulates BDNF expression in the mouse brain by a mechanism similar to the one induced by exercise. Indeed, physical exercise increases BHB levels and BDNF expression in the hippocampus [84]. BDNF expression is increased after an intraventricular infusion of BHB [85]; thus, the increased level of blood BHB reached during a ketogenic diet [9,86] may explain the increased BDNF in the KD group. We have already observed no negative mood variations during KD [87] and an increased BDNF level may explain this observation; moreover, a higher level of BDNF may improve cognition and memory, important factors in athletes.

## 5. Limitations

One of the limitations of this study was the impossibility of standardizing training. Participants were all experienced athletes with many years of training experience; all performed from three to four sessions per week, but the volume and the intensity of the workouts were decided by each individual. The competitive season always takes place around the same time of the year, and the athletes were recruited and started the study in the same period. Therefore, given that the study was conducted for all subjects at the same time of the year (off-season), the training program was similar for participants and aimed at improving muscle mass [21,88,89]. We should also point out that this research studied expert bodybuilders, and thus not all the results may be generalized to other sports or the general population.

## 6. Conclusions

The results show that a KD diet may represent an adequate dietary approach for BB athletes. Despite the lack of hypertrophic response in the KD group, muscle mass was maintained, a phenomenon that often does not occur during low-calorie diets. Similarly, although the time of year was not the one that athletes usually dedicate to training for fat loss (“cutting”), KD proved to be a good strategy to reduce body fat.

KD also resulted in a decrease in inflammatory cytokines and the increase in BDNF, suggesting that KD can be a valid tool for dealing with moments (such as that of “weightlifting”) where stress management and maintenance of motivation are hard to handle. KD is not a regime to be followed lightly and independently but requires the presence of a professional; in these circumstances KD represents a fundamental tool in the nutritionist’s baggage to face various conditions and needs, including those of sports.

## Figures and Tables

**Table 1:** Baseline characteristics of Ketogenic Diet (KD) and control western diet (WD) groups.

	KD (*n* = 9)	WD (*n* = 10)	*p*-Value
Age (y)	26.22 ± 5.09	31.67 ± 10.39	0.16
Weight (kg)	86.39 ± 15.42	89.04 ± 11.73	0.68
BMI (kg/m^2^)	26.97 ± 1.86	26.66 ± 2.04	0.73
Lean mass (%)	88.88 ± 2.66	88.38 ± 3.06	0.71

All values are means ± SD. KD, ketogenic diet group; WD, western diet group.

**Table 2:** Diet composition and macronutrients distribution.

	KD (*n* = 9)	WD (*n* = 10)
Total Energy intake (kcal/day)	3443.70 ± 545.94	3529.71 ± 374.06
Protein (kcal)	863.89 ± 154.19	890.40 ± 117.30
Carbohydrates (kcal)	175.00 ± 28.17	1952.50 ± 209.43 *
Fat (kcal)	2379.81 ± 393.78	707.10 ± 63.66 *
Protein (g)	215.97 ± 38.55	222.60 ± 29.33
Carbohydrates (g)	43.75 ± 7.04	488.13 ± 52.36 *
Fat (g)	264.42 ± 43.75	78.57 ± 7.07 *
Protein (%)	24.65 ± 1.24	25.03 ± 0.91
Carbohydrates (%)	5.00 ± 0.00	55.00 ± 0.00 *
Fat (%)	68.00 ± 2.27	19.97 ± 0.91 *

All values are means ± SD. * significantly different from KD group (*p* < 0.05). KD, ketogenic diet group; WD, western diet group.

**Table 3:** Muscle strength and basal metabolism results.

	KD (*n* = 9)		WD (*n* = 10)		Diet *p*-Value	Time *p*-Value	Time × Diet *p*-Value
	Pre	Post	Pre	Post			
Bench press 1 RM (kg)	129.78 ± 20.98	134.44 ± 17.14 *	136.40 ± 11.27	141.40 ± 10.24 *	ns	0.0009	ns
Squat 1 RM (kg)	181.33 ± 36.52	187.78 ± 37.41 *	176.10 ± 27.87	187.00 ± 26.96 *	ns	<0.0001	ns
Basal metabolism (REE) (Kcal/day)	2014.67 ± 324.04	2052.56 ± 317.99	2069.10 ± 229.32	2125.20 ± 206.08 *	ns	0.0006	ns
Respiratory exchange ratio (RER)	0.82 ± 0.01	0.79 ± 0.02 *	0.83 ± 0.01	0.83 ± 0.02 #	0.0022	0.0002	0.0001

All values are means ± SD. * significantly different from pre value (*p* < 0.05); # significantly different from KD group (*p* < 0.05). KD, ketogenic diet group; WD, western diet group.

**Table 4:** Blood parameters.

	KD (*n* = 9)		WD (*n* = 10)		Diet *p*-Value	Time *p*-Value	Time × Diet *p*-Value
	Pre	Post	Pre	Post			
Lipid profile							
Total Cholesterol (mg/dL)	194.78 ± 8.88	187.89 ± 10.15 *	193.90 ± 18.18	190.60 ± 16.73	ns	0.0071	ns
HDL (mg/dL)	57.22 ± 3.33	60.00 ± 3.33 *	52.50 ± 6.11	51.80 ± 5.05 #	0.0072	ns	0.0039
LDL (mg/dL)	113.33 ± 8.88	108.00 ± 10.28	118.60 ± 20.64	116.20 ± 18.39	ns	ns	ns
TG (mg/dL)	121.00 ± 26.70	99.22 ± 19.72 *	114.70 ± 13.21	112.70 ± 13.06	ns	<0.0001	0.0003
Transaminase							
Aspartate transaminase (AST) (mg/dL)	38.78 ± 2.82	38.44 ± 2.54	39.10 ± 3.67	39.30 ± 4.11	ns	ns	ns
Alanine amino transferase (ALT) (mg/dL)	43.11 ± 6.51	38.56 ± 3.30 *	42.10 ± 6.59	44.80 ± 5.92	ns	ns	0.0086
Anabolic Hormones							
Testosterone total (nmol/L)	21.76 ± 5.33	19.32 ± 4.09 *	20.96 ± 5.13	21.27 ± 4.91	ns	0.0094	0.0016
IGF-1 (ng/mL)	213.33 ± 39.41	181.50 ± 25.93 *	222.40 ± 34.27	219.80 ± 23.42 #	ns	0.0050	0.0124

All values are means ± SD. * significantly different from pre value (*p* < 0.05); # significantly different from KD group (*p* < 0.05). KD, ketogenic diet group; WD, western diet group.

## Data Availability

Data available on request from the authors.
